# A rapid chemical method for lysing *Arabidopsis *cells for protein analysis

**DOI:** 10.1186/1746-4811-7-22

**Published:** 2011-07-15

**Authors:** Daisuke Tsugama, Shenkui Liu, Tetsuo Takano

**Affiliations:** 1 Asian Natural Environmental Science Center (ANESC), The University of Tokyo, 1-1-1 Midori-cho, Nishitokyo-shi, Tokyo, 188-0002, Japan; 2 Alkali Soil Natural Environmental Science Center (ASNESC), Northeast Forestry University, Harbin 150040, PR China

**Keywords:** Cell wall, pectin, Ca^2+^, Chelate, detergent, *Arabidopsis*

## Abstract

**Background:**

Protein extraction is a frequent procedure in biological research. For preparation of plant cell extracts, plant materials usually have to be ground and homogenized to physically break the robust cell wall, but this step is laborious and time-consuming when a large number of samples are handled at once.

**Results:**

We developed a chemical method for lysing *Arabidopsis *cells without grinding. In this method, plants are boiled for just 10 minutes in a solution containing a Ca^2+ ^chelator and detergent. Cell extracts prepared by this method were suitable for SDS-PAGE and immunoblot analysis. This method was also applicable to genomic DNA extraction for PCR analysis. Our method was applied to many other plant species, and worked well for some of them.

**Conclusions:**

Our method is rapid and economical, and allows many samples to be prepared simultaneously for protein analysis. Our method is useful not only for *Arabidopsis *research but also research on certain other species.

## Background

Protein extraction is a frequent procedure in biological research. For preparation of plant cell extracts, it is usually required to grind and homogenize plant materials to physically break the robust cell wall. Sample grinding is laborious and time-consuming, especially when a large number of samples are handled at once. In the case of yeast cells, which also have a cell wall, proteins can be efficiently extracted after cells are treated with alkaline (NaOH) and boiled in SDS-containing solution [1-3]. Although the components and structure of the yeast cell walls [4, for a review] are different from those of the plant cell wall [5, for a review], the simplicity of the yeast method tempted us to seek out a chemical cell-lysis method suitable for plant protein extraction.

Here we describe a rapid and simple way of preparing cell extracts from *Arabidopsis*. We found that in the presence of certain amounts of a Ca^2+ ^chelator and detergent, *Arabidopsis *cells are quickly lysed just by boiling, without grinding. The method is rapid and economical, and the cell extracts prepared by the method are suitable for SDS-PAGE and immunoblot analysis.

## Results and Discussion

### Effects of Ca^2+^-chelating agents on *Arabidopsis *cell lysis

**Figure 1:**
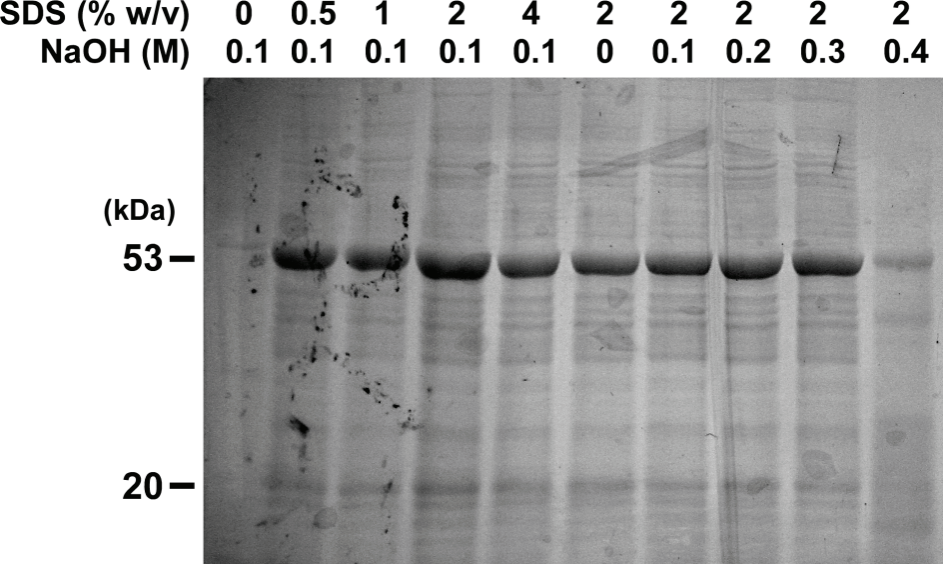
**Chemical lysis of *Arabidopsis *cells**. Three 7-day-old *Arabidopsis *seedlings were boiled for 10 minutes in the 100 μl lysis solution (100 mM Tris-HCl, pH 8.0, and indicated concentrations of SDS and NaOH). The solution was then mixed with 2× Laemmli sample buffer in 1:1 volume, and subjected to SDS-PAGE followed by CBB staining.

**Figure 2:**
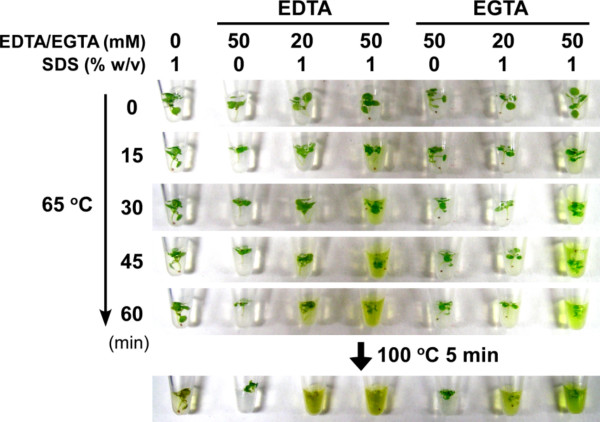
**EDTA/EGTA-dependent *Arabidopsis *cell lysis**. A 7-day-old *Arabidopsis *seedling was placed in the lysis solution (100 mM Tris-HCl, pH 8.0, and the indicated concentrations of SDS and EDTA/EGTA), incubated at 65°C for 60 minutes, and then at 100°C for 5 minutes. Photographs were taken at the indicated time points.

**Figure 3:**
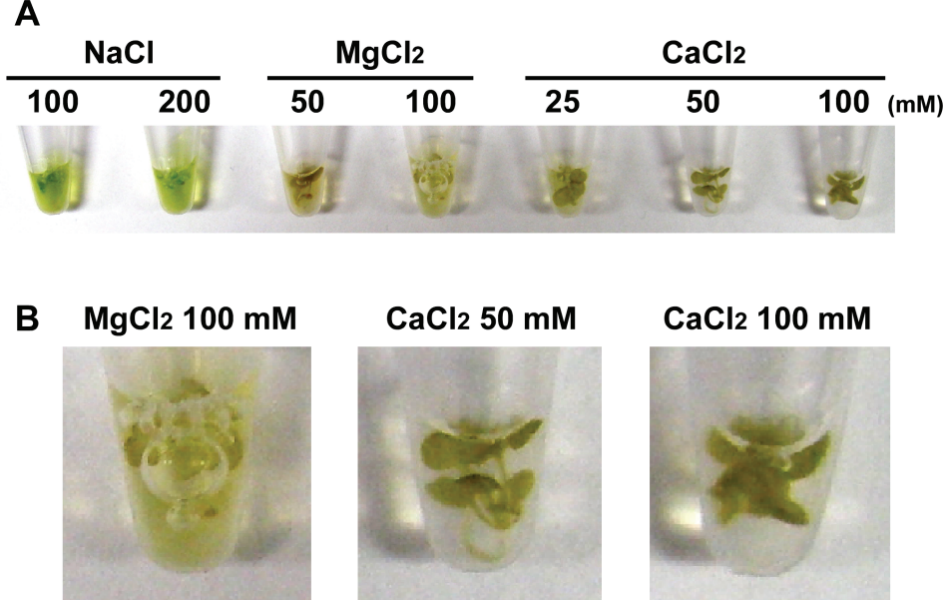
**Effects of cations on EDTA/EGTA-dependent *Arabidopsis *cell lysis**. (A) A 7-day-old *Arabidopsis *seedling was put in the lysis solution (50 mM EDTA, 1% w/v SDS, 100 mM Tris-HCl, pH 8.0, and the indicated concentrations of NaCl, MgCl2 or CaCl2), and incubated at 100°C for 10 minutes. (B) Enlarged images of those shown in panel A.

In an effort to develop a rapid method of sample preparation, we first simply applied the yeast "alkaline lysis" procedure [3] to *Arabidopsis *seedlings. Plants were incubated at 100°C for 10 minutes in the solution containing SDS, NaOH, EDTA and β-ME (β-mercaptoethanol). After the treatment, the solution turned green due to chlorophyll leakage from the cells. SDS-PAGE followed by CBB (Coomassie brilliant blue) protein staining confirmed that cytoplasmic proteins were also present in the supernatant, suggesting that this method works well in protein extraction from *Arabidopsis*. Because having the proper concentrations of SDS and NaOH in the solution was critical in the yeast method [1-3], we examined the effects of different concentrations of SDS and NaOH on protein extraction from *Arabidopsis*. SDS was indispensable for efficient protein extraction, but unexpectedly, the NaOH concentration had little effect on the efficiency. When a higher (0.4 M) concentration of NaOH was used, less amounts of protein were detected (Figure 1). This was probably due to protein degradation but not to insufficient protein extraction, because the plant cells lost their green color and appeared to be lysed even when the NaOH concentration was 0.4 M (data not shown). The NaOH-free solution (containing SDS, EDTA and β-ME) worked well, while in preliminary experiments, 2× Laemmli sample buffer (containing SDS, and β-ME but no EDTA) [6] failed to extract proteins from unground plants. This suggested that EDTA was necessary for efficient protein extraction, and thus we examined the effects of EDTA on cell wall loosening. We heated plants in the solution containing SDS and EDTA, and saw greening of the solution by leakage of chlorophyll, the molecular weight of which is smaller than those of usual proteins, as a sign of cell wall loosening. Chlorophyll leakage increased with increasing incubation period, increasing temperature and increasing EDTA concentration. A Ca^2+^-specific chelator, EGTA, was as effective as EDTA in extracting chlorophyll (Figure 2), suggesting that EDTA/EGTA-Ca chelate formation is involved in the cell lysis process. EDTA chelates Mg^2+ ^or Ca^2+ ^in a molar ratio of 1:1. In the presence of MgCl_2_, chlorophyll leakage was retarded but occurred to some extent, whereas excess CaCl_2 _strongly suppressed chlorophyll leakage. NaCl did not affect the efficiency of chlorophyll leakage (Figure 3). These results provide further evidence that chelating Ca^2+ ^in the cell wall is important for efficient cell lysis. Unrestricted diffusion of molecules through the cell wall is limited by the pore size of the cell wall, and the pore size of the primary cell wall is defined by pectins [5, for a review]. Ca^2+ ^is a component of pectins, and stabilizes their structure [5, for a review]. Thus our results can be attributed to pectin solubilization by EDTA/EGTA. Cells were not lysed well when SDS treatment was followed by EDTA treatment and vice versa (data not shown), which suggests that cell lysis requires simultaneous cell wall loosening by EDTA/EGTA and membrane solubilization by SDS.

**Figure 4:**
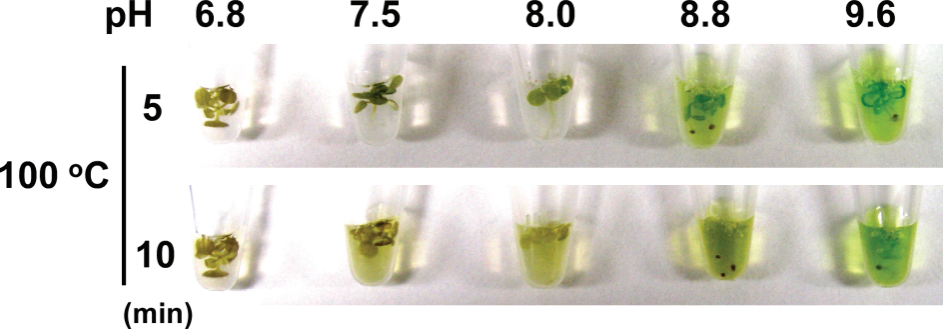
**pH-dependent *Arabidopsis *cell lysis**. A 7-day-old *Arabidopsis *seedling was put in the lysis solution (1% w/v SDS and 200 mM Tris-HCl of the indicated pH), and incubated at 100°C for 10 minutes. Photographs were taken at the indicated time points.

We also examined effects of pH on cell wall loosening using Tris-HCl buffers with pH values from 6.8 to 9.6. We found that chlorophyll could be efficiently extracted without EDTA when pH was 8.8 or 9.6 (Figure 4). This finding is consistent with the previous studies reporting that DNA can be extracted after plants are treated by NaOH [7,8] or Tris-HCl, pH 9.5 [9]. When pH was below 8.8, chlorophyll extraction was incomplete without EDTA (Figure 4). Because an EDTA/EGTA-Ca chelate is more stable when pH of the solution is higher [10], we compared the efficiencies of chlorophyll extraction at pH 6.8, 7.5 and 8.0 in the presence of EDTA. The efficiency depended on the EDTA concentration rather than on pH or the stability of the EDTA-Ca chelate (data not shown).

The effects of EDTA and EGTA and five other Ca^2+ ^chelators on *Arabidopsis *cell lysis were compared. All of the chelators were effective at a concentration of 50 mM, while some had little or no effect at 20 mM (Table 1). The effectiveness did not appear to be correlated with the chelate stability. We examined four other detergents besides SDS: an anionic detergent, SDSa (sodium N-dodecanoylsarcosinate), a zwitterionic detergent, CHAPS (3-[(3-cholamidopropyl)dimethylammonio]propane- sulfonate), and two nonionic detergents, Tween 20 and Brij 35. All except Tween-20 caused the cell lysis in combination with EDTA (data not shown). Thus, *Arabidopsis *cells can be rapidly lysed if they are boiled in the presence of an appropriate Ca^2+ ^chelator and detergent.

**Table 1:** Chelators used in this study

		Cell lysis*^2^	
		
Chelator	logK_CaL_*^1^	20 mM	50 mM
EDTA	10.96	+	+
EGTA	11	+	+
NTA	6.41	-	+
TTHA	10.06	-	+
DTPA	10.74	-	+
CyDTA	12.5	±	+
BAPTA	6.97	+	+

*^1 ^Chelate-stability constants, which are available in the Dojindo website http://www.dojindo.co.jp/.

*^2 ^A 7-day-old *Arabidopsis *seedling was boiled for 10 min in the 50 - l lysis solution (100 mM Tris-HCl, pH 8.0, 1% w/v SDS and 20 or 50 mM chelator listed). Samples were regarded as lysed (+) or not (-), based on the development of green color of the chlorophyll in the solution.

### Rapid preparation of cell extracts

**Figure 5:**
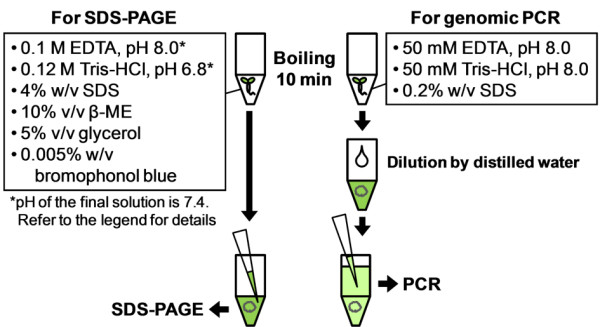
**Diagram of rapid sample preparation for SDS-PAGE and genomic PCR analysis from *Arabidopsis***. The lysis solution for SDS-PAGE was prepared by mixing stock solutions of its components (0.5 M EDTA, pH 8.0, 1 M Tris-HCl, pH 6.8, 10% w/v SDS, 100% β-ME, 100% glycerol and bromophenol blue powder). The pH value of this solution was around 7.4. The lysis solution for genomic PCR was prepared in the same way. *Arabidopsis *cells can be rapidly lysed by boiling plants in the indicated solutions for 5-10 minutes (until the solution become cloudy by cell extracts). In the case of SDS-PAGE, the solution can be directly loaded onto the gel after boiling. In the case of genomic PCR, the solution is diluted after boiling (500 μl distilled water for 50 μl lysis solution) and usable as a PCR template.

**Figure 6:**
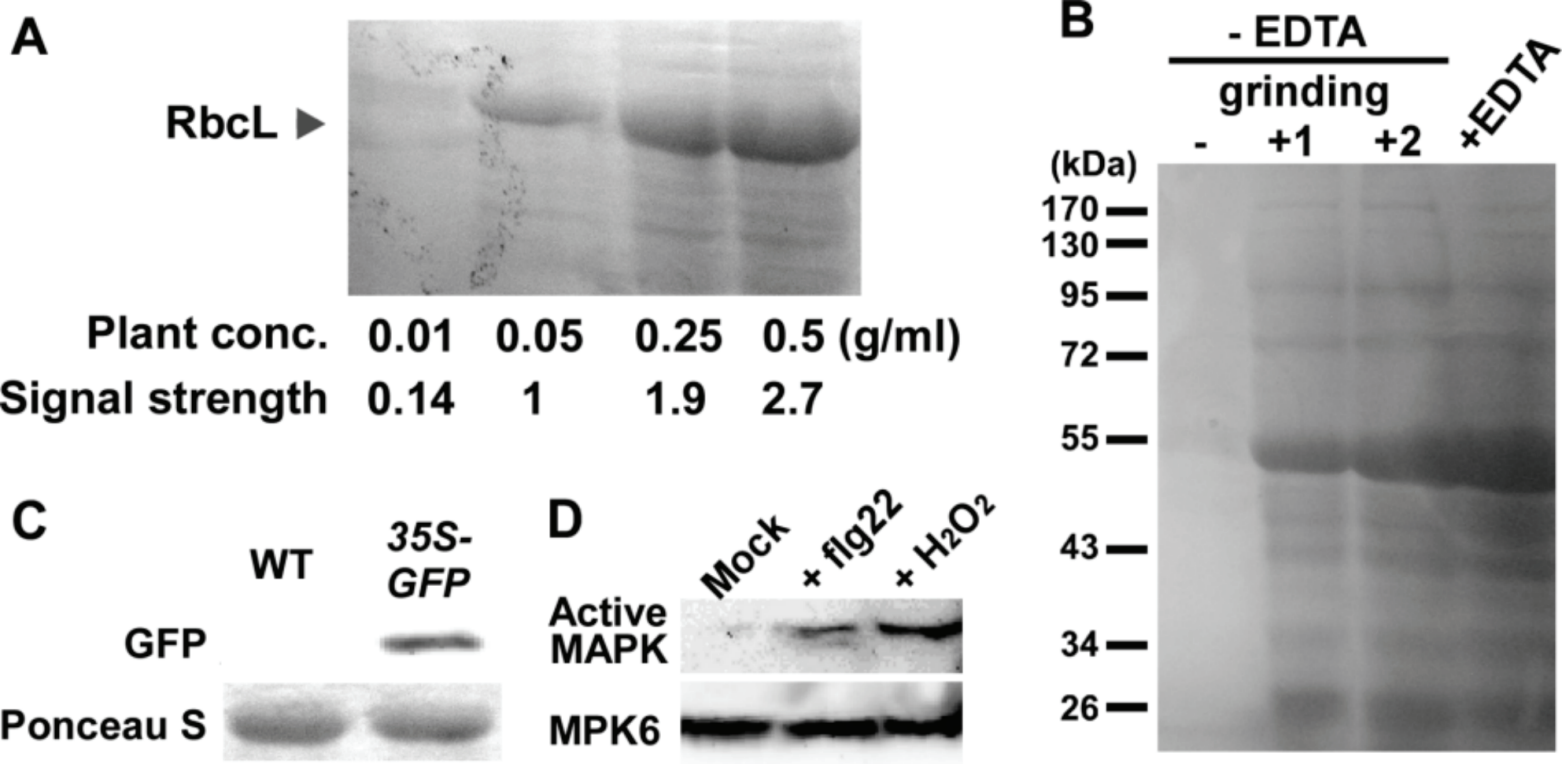
**Compatibility of the chemical cell lysis method for protein analyses**. Protein samples were prepared by the procedure shown in Figure 5 unless otherwise stated. (A) SDS-PAGE analysis of proteins extracted by the chemical method. *Arabidopsis *seedlings were boiled in the lysis solution at the indicated concentrations (Plant conc.). The arrowhead indicates the position of Rubisco large subunit (RbcL). Signal intensities of RbcL are shown as relative values (Signal strength). (B) Comparison of the chemical method with usual grinding methods. For +EDTA, 50 mg leaves of 3-week-old *Arabidopsis *were boiled in 100 μl of the lysis solution as shown in Figure 5. For - EDTA, grinding +1, 50 mg leaves were ground with a pestle homogenizer in 100 μl of 2× Laemmli sample buffer and boiled. For - EDTA, grinding +2, 50 mg leaves were frozen in liquid nitrogen, ground to powder using a mortar and pestle, and boiled in 100 μl of 2× Laemmli sample buffer. For - EDTA, grinding -, 50 mg unground leaves were boiled in 2× Laemmli sample buffer. (C) Immunoblot analysis of GFP. Samples were prepared from wild-type plants (WT) and transgenic plants expressing GFP under the control of the CaMV35S promoter (*35S-GFP*). Proteins on the membrane were stained with Ponceau S to ensure equal protein loading (bands of RbcL are shown). (D) Immunoblot analysis of flg22- or H2O2-induced MAPK activation. Seedlings were treated with 1 μM flg22 (+ flg22) or 40 mM H2O2 (+ H2O2) and subjected to the immunoblot analysis.

Although *Arabidopsis *cell walls can be loosened with either a Ca^2+ ^chelator or high pH, highly alkaline conditions can degrade proteins (Figure 1). Moreover, we observed protein degradation when plants were boiled in a solution of pH 9.6 after H_2_O_2 _treatment, but not when pH 6.8 (data not shown). Therefore we adopted an EDTA-dependent cell lysis method. The sample preparation procedure is illustrated in Figure 5. In this system, samples can be loaded after just 10 minutes of boiling in the lysis buffer, allowing many samples to be prepared simultaneously. Plant materials up to 0.5 g/ml could be almost completely lysed in our lysis solution (Figure 6A). Using our method or usual grinding methods, we extracted proteins from the same amount (50 mg) of plant tissues and compared the protein banding patterns. The protein banding pattern obtained through our method was similar to those obtained through the usual grinding methods (Figure 6B), suggesting that our method is as efficient as the usual grinding methods in extracting proteins. We performed immunoblot analyses as practical applications of our method. Using samples prepared by our system, we successfully detected GFP in transgenic plants and MAPK (mitogen-activated protein kinase) activation induced by the flg22 [11] or H_2_O_2 _treatment [12] (Figure 6B, C). In either case, degradation products were not detected (data not shown). These results confirm that our method is compatible for analysis of transgene products and physiological responses.

**Figure 7:**
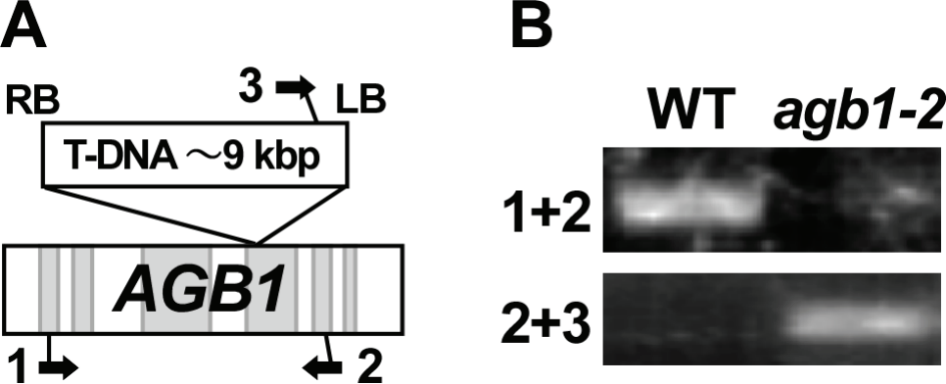
**Compatibility of the chemical method for genomic DNA analysis**. (A) Diagram of T-DNA insertion in *AGB1*. Exons are shown as light gray boxes. Arrows 1, 2 and 3 represent *AGB1 *forward primer, *AGB1 *reverse primer and T-DNA left border primer, respectively. RB: right border; LB: left border. (B) PCR templates were prepared by the procedure shown in Figure5 from wild-type (WT) and *agb1-2 *mutant plants. Primer pairs used for PCR are shown as 1+2 and 2+3 (the numbers correspond to those in panel A).

We also applied our method to genomic PCR analysis. PCR template was prepared by the procedure shown in Figure 5 from wild-type plants and *agb1-2 *mutant plants, which have a T-DNA insertion in *AGB1 *[13]. A DNA fragment of *AGB1 *could be amplified from the wild-type template but not from the *agb1-2 *template when the *AGB1*-specific primer pair was used. On the other hand, a DNA fragment was amplified only from the *agb1-2 *template when a T-DNA-specific primer was used (Figure 7). Our sample preparation method is simple and quick, but without further purification of DNA, a contaminant-tolerant PCR enzyme must be used. We tried several kinds of PCR enzymes, and KOD FX gave the best results (data not shown). An excess amount of EDTA or SDS interferes with PCR. There might be a less inhibitory combination of Ca^2+ ^chelator and detergent, though changing detergents from anionic SDS to nonionic Brij 35 did not improve the PCR efficiency in our experiment (data not shown).

### Applicability of the method to different tissues and plants

**Figure 8:**
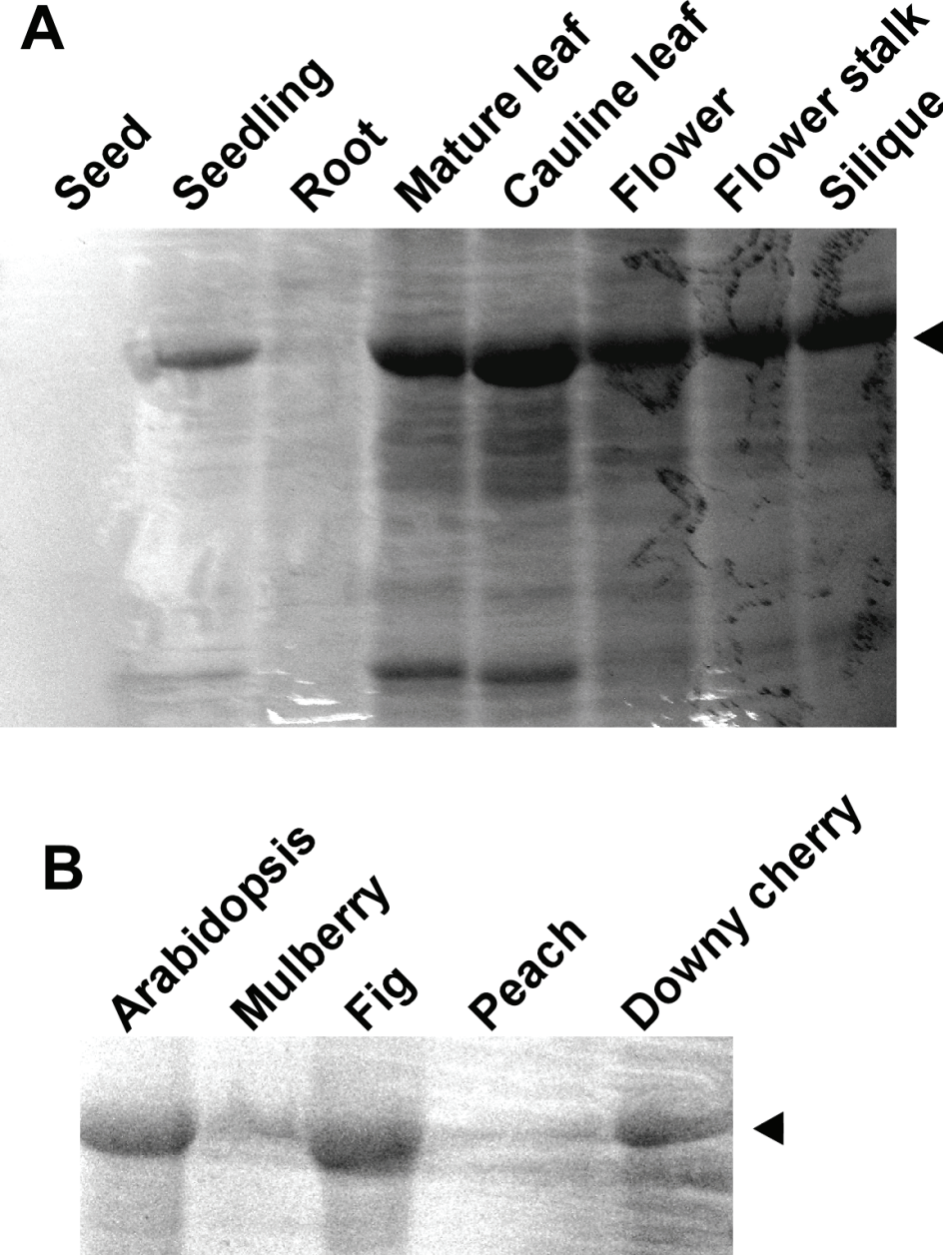
**Application of the chemical cell lysis method to various tissues of *Arabidopsis *(A) and to leaves of other plant species (B)**. (A) Indicated tissues of *Arabidopsis *(0.1-0.5 g/ml in concentrations) were subjected to the chemical lysis described in Figure 5 and then to SDS-PAGE. Proteins were visualized by CBB staining. The arrow head indicate the position of Rubisco large subunit (RbcL). (B) Protein samples were prepared by the method described in Figure 5 from leaves of mulberry (*Morus bombycis*), fig (*Ficus carica*), peach (*Amygdalus persica*) and downy cherry (*Prunus tomentosa*) as well as from leaves of *Arabidopsis*. Plants were lysed in approximately 0.25 g/ml concentrations. The samples were subjected to SDS-PAGE followed by CBB staining. The arrowhead indicates the position of RbcL.

Our method does not work on all plant tissues or species. Proteins could be extracted from most *Arabidopsis *tissues, but not from seeds, which are covered with robust seed coats (Figure 8A). The extraction method worked well for leaves of fig (*Ficus carica*) and downy cherry (*Prunus tomentosa*) (Figure 8B), but not for leaves of mulberry (*Morus bombycis*) or peach (*Amygdalus persica*), possibly because their cell walls are more resistant to the lysis solution. Silver grass (*Miscanthus sinensis*) and many monocot wild plants could not be lysed by our method, even if the boiling treatment was prolonged (data not shown). This may be because the latter plants belong to the family Poaceae or Cyperaceae, which has a special primary cell wall called a type II wall, which is chemically and structurally different from the cell walls of most dicots as well as some monocots [5, for a review].

## Conclusions

We have developed a chemical method for lysing unground *Arabidopsis *cells, which is based on cell wall loosening by a Ca^2+ ^chelator and on membrane solubilization by detergent. Our method is applicable to some other species, and cell extracts prepared by our method is suitable for SDS-PAGE, immunoblot and genomic PCR analysis. Our method is rapid, economical, and thus useful for plant research.

## Methods

### Chemicals

EDTA (EDTA disodium salt dihydrate) was purchased from Bio-Rad (Japan), EGTA from Sigma (USA), NTA (nitrilotriacetic acid), TTHA (triethylenetetramine-*N, N, N', N", N"', N"'*-hexaacetic acid), DTPA (diethylenetriamine-*N, N, N', N", N"*-pentaacetic acid), CyDTA (*trans*-1,2-diaminocyclohexane-*N, N, N', N'*-tetraacetic acid, monohydrate) and BAPTA (*O,O*'-bis(2-aminophenyl)ethyleneglycol-*N, N, N', N'*-tetraacetic acid, tetrapotassium salt, hydrate) from Dojindo (Japan). All these chelating agents except EDTA were prepared as 100 mM stock solutions by diluting them in 0.4 M NaOH. EDTA was prepared as a 0.5 M stock solution of pH 8.0.

SDS, SDSa and Tween 20 were purchased from Wako (Japan), CHAPS from Dojindo, and Brij 35 from Calbiochem. SDS, SDSa and CHAPS were prepared as 10% w/v stock solutions in distilled water, Tween 20 and BriJ 35 as 10% v/v. SDS was used in the concentrations described in the figures. SDSa and CHAPS were used in the final concentrations of 0.1-1% w/v. Tween 20 and Brij 35 were used in the final concentration of 0.1-1% v/v.

### Plant materials, growth conditions and plant transformation

*Arabidopsis thaliana *ecotype Columbia-0 (Col-0) was used throughout the experiments. Seeds of *agb1-2 *were obtained from the Arabidopsis Biological Resource Center (ABRC, http://www.arabidopsis.org). Surface-sterilized seeds were sown on the 0.5× MS medium (0.8% w/v agar, 0.5× MS salts, 1% w/v sucrose, 0.5 g/l MES, pH 5.8) for immunoblot and genomic PCR analyses, or in rockwool cubes for the other experiments. In either case, plants were grown at 22°C under a 12-h light/12-h dark photoperiod. To generate transgenic plants expressing GFP, the GUS coding sequence in pBI121 was replaced with the GFP coding sequence, and this construct was used for *Arabidopsis *transformation by the *Agrobacterium*-mediated floral-dip method [14]. GFP expression in T2 plants was checked by fluorescence microscopy, and GFP-positive plants were used for the immunoblot analysis. Plants other than *Arabidopsis *were grown in the field and healthy-looking leaves were sampled.

### Preparation of *Arabidopsis *cell extracts

All the compositions of the solutions and the detailed procedures for the cell lysis experiments are described in each figure. All the lysis solutions were made by mixing stock solutions of their components. For example, the lysis solution for SDS-PAGE in Figure 5 (0.1 M EDTA, 0.12 M Tris-HCl, 4% w/v SDS, 10% v/v β-ME, 5% v/v glycerol and 0.005% w/v bromophenol blue) was prepared by mixing 0.5 M EDTA, pH 8.0, 1 M Tris-HCl, pH 6.8, 10% w/v SDS, 100% β-ME, 100% glycerol and bromophenol blue powder to give the final concentrations. Images were processed with Canvas X software (ACD Systems).

### SDS-PAGE, immunoblot analysis and genomic PCR analysis

SDS-PAGE and immunoblot analysis were carried out according to standard procedures [6,15]. After CBB staining, signals strengths were quantified using ImageJ software http://rsb.info.nih.gov/ij/index.html. For GFP detection, 7-day-old seedlings grown on the 0.5× MS medium were used. For detection of MAPK activation, 14-day-old seedlings were incubated at room temperature for 20 min in 20 mM Tris-HCl, pH 6.8 with or without 1 μM flg22 or 40 mM H_2_O_2_. Immunoblot analysis was performed using an anti-GFP (MBL, Japan), anti-MPK6 (Sigma) or anti-phospho-p44/42 MAPK (Erk1/2) (Cell Signaling Technology, USA) as a primary antibody, and a horseradish peroxidase-conjugated anti-rabbit IgG (MBL) as the secondary antibody. Signals were detected using SuperSignal West Pico Chemiluminescent Substrate (Thermo Fisher Scientific, USA) and LAS-1000 plus image analyzer (Fuji Film, Japan). After detecting GFP, total proteins on the membrane were stained with Ponceau S solution (0.2% w/v Ponceau S in 5% acetic acid).

For genomic PCR analysis, genomic DNA was prepared from 7-day-old seedlings by the procedure described in Figure 5. PCR was performed using KOD FX (Toyobo, Japan). For 50 μl PCR mixture, 1-3 μl of the template solution was added. Primer sequences were as follows: AGB1 forward, 5'-AGACGCCTCCAGCTCCTCGA-3'; AGB1 reverse, 5'-GCACTTCCATCTGCTGACAACCCC-3'; T-DNA left border, 5'-CAGGATTTTCGCCTGCTGGGGC-3'. PCR cycle: 98°C 2 min, 40 cycles of (98°C 10 sec, 60°C 30 sec, 72°C 1 min), 72°C 7 min, 4°C until analysis. Images were processed with Canvas X software (ACD Systems).

## Abbreviations

SDS: sodium dodecyl sulfate; PAGE: polyacrylamide-gel electrophoresis; EDTA: ethylenediaminetetraacetic acid; --ME: β-mercaptoethanol; CBB: Coomassie brilliant blue; EGTA: ethyleneglycol-bis(β-aminoethyl)tetraacetatic acid; SDSa: sodium N-dodecanoylsarcosinate; CHAPS: 3-[(3-cholamidopropyl)dimethylammonio]propane- sulfonate; GFP: green fluorescent protein; MAPK: mitogen-activated protein kinase; NTA: nitrilotriacetic acid; TTHA: triethylenetetramine-*N, N, N', N", N"', N"'*-hexaacetic acid; DTPA: diethylenetriamine-*N, N, N', N", N"*-pentaacetic acid; CyDTA (*trans*-1,2-diaminocyclohexane-*N, N, N', N'*-tetraacetic acid; BAPTA: *O, O*'-bis(2-aminophenyl)ethyleneglycol-*N, N, N', N'*-tetraacetic acid; MS: Murashige and Skoog; MES: 2-(4-Morpholino)ethane sulfonic acid.

## Competing interests

The authors declare that they have no competing interests.

## Authors' contributions

DT designed the experiments. DT and SL performed the experiments. DT and TT wrote the manuscript. All authors read and approved the final manuscript.
